# Amygdala, Medial Prefrontal Cortex and Glucocorticoid Interactions Produce Stress-Like Effects on Memory

**DOI:** 10.3389/fnbeh.2019.00210

**Published:** 2019-09-18

**Authors:** Eun Joo Kim, Jeansok J. Kim

**Affiliations:** ^1^Department of Psychology, University of Washington, Seattle, WA, United States; ^2^Program in Neuroscience, University of Washington, Seattle, WA, United States

**Keywords:** stress, corticosterone, amygdala, prefrontal cortex, hippocampus, object recognition memory, learning, cognition

## Abstract

Adverse stress effects on the hippocampal memory system are generally thought to be due to the high level of circulating glucocorticoids directly modifying the properties of hippocampal neurons and, accordingly, the results should be reproducible with exogenous administration of cortisol in humans and corticosterone in rodents. However, glucocorticoid levels increased to other events, such as exercise and environment enrichment, do not impair but instead enhance hippocampal memory, indicating that cortisol/corticosterone are not invariant causal factors of stress. To better model the complex psychophysiological attributes of stress (i.e., aversiveness, lack of controllability, and glucose metabolism), we examined the functions of the amygdala, medial prefrontal cortex (mPFC), and corticosterone on a hippocampal-based one-trial novel object recognition (OR) memory task in rats. Specifically, animals were subjected to amygdala stimulation, mPFC inactivation, and corticosterone treatments separately or in combination during behavioral testing. Collective amygdala, mPFC, and corticosterone manipulations significantly impaired OR memory comparable to behavioral stress. By contrast, single and dual treatments failed to reliably decrease memory functioning. These results suggest that negative mnemonic impacts of uncontrollable stress involve the amalgamation of heightened amygdala and diminished mPFC activities, and elevated circulating corticosterone level.

## Introduction

The hypothalamic-pituitary-adrenal (HPA) axis hormones are widely thought to play necessary and sufficient roles in producing various detrimental outcomes of uncontrollable stress (Sapolsky, 2000; McEwen, 2013). Among the brain structures, the hippocampus and its mnemonic functions are deemed particularly sensitive to stress because hippocampal cells pack high concentration of receptors for corticosteroids (glucocorticoids and mineralocorticoids), whose synthesis and secretion by the adrenal cortex are augmented by stress (McEwen and Sapolsky, 1995; Kim et al., 2015). In support of this view, human and animal studies have revealed an inverted-U functional relationship between the level of circulating glucocorticoids and the performance of declarative-explicit memory tasks (Lupien and Lepage, 2001; Kim and Diamond, 2002; Het et al., 2005; Kim et al., 2015). For example, patients with Cushing’s syndrome, a hypercortisolemia condition in which tumors affect the HPA axis, and healthy individuals who are administered high doses of cortisol have subpar performance in verbal recall tasks (Starkman et al., 1992; Newcomer et al., 1994). Similarly, rodents injected with corticosterone underperform in spatial memory tasks (de Quervain et al., 1998; Coburn-Litvak et al., 2003). Furthermore, animal studies have implicated corticosterone in altering long-term synaptic plasticity (Pavlides et al., 1996), decreasing dendritic arborization (Woolley et al., 1990; Morales-Medina et al., 2009), suppressing adult neurogenesis (Oomen et al., 2007; Brummelte and Galea, 2010), and even causing necrosis (Masters et al., 1989) in the hippocampus. These neurophysiological changes have been proposed to occur through elevated levels of corticosteroids saturating the lower-affinity *Type-II* glucocorticoid, as opposed to the high-affinity *Type-I* mineralocorticoid receptors (Pavlides et al., 1995; Yang et al., 2004; Oomen et al., 2007). Clinically, there are reports of both increased (Pitman and Orr, 1990; Lemieux and Coe, 1995; Maes et al., 1998) and decreased (King et al., 2001; Oquendo et al., 2003; Yehuda et al., 2005) levels of glucocorticoids in stress-induced psychopathologies, namely posttraumatic stress disorder (PTSD).

Although numerous studies ascribe memory-impairing effects of stress solely in relation to cortisol (in humans) and corticosterone (in rodents) levels (Starkman et al., 1992; Newcomer et al., 1994; McEwen and Sapolsky, 1995; Heinrichs et al., 1996; de Quervain et al., 1998), there is conflicting evidence that glucocorticoids alone cannot reproduce behavioral stress effects on the hippocampus. For example, exercise and environmental enrichment substantially increase corticosterone levels, but they enhance, rather than impair, hippocampal memory and neurogenesis (Kempermann et al., 2002; Hötting et al., 2016; Kim et al., 2018). Likewise, male rats exposed to receptive females show intact spatial working memory despite having significantly elevated plasma corticosterone levels equivalent to stress-induced levels (Woodson et al., 2003). Animal studies have also demonstrated a double dissociation between corticosterone and hippocampal functions. Specifically, stress continues to impair hippocampal long-term potentiation (LTP) in adrenalectomized rats depleted of corticosterone (Diamond et al., 1992), and amygdala lesion/inactivation block stress impairment of LTP and spatial memory without impeding stress enhancement of corticosterone levels (Kim et al., 2001, 2005). The medial prefrontal cortex (mPFC) has also been found to mitigate stress-induced learned helplessness *via* inhibiting the dorsal raphe nucleus (DRN; Amat et al., 2005) and regulate the HPA axis hormone responses to stress (Diorio et al., 1993). Correspondingly, the mPFC has been reported to be volumetrically smaller and hyporesponsive in PTSD patients (Shin et al., 2001). It appears then the cognitive-affective-arousal reactivity aspects of uncontrollable stress require a systems-level, rather than glucocorticoids-centered, analysis. Hence, the present study examined, for the first time, the ensemble functions of the amygdala (AMYG; concerned with affective responses), mPFC (implicated in top-down cognitive control), and corticosterone (CORT; indicative of heightened arousal and glucose metabolism) in generating stress effects on hippocampal-based one-trial novel object recognition (OR) memory in rats (Ennaceur and Delacour, 1988; Clark et al., 2000; Baker and Kim, 2002).

## Materials and Methods

### Ethics Statement

All experiments were performed in compliance with the NIH Guide for the Care and Use of Laboratory Animals and under protocols approved by the University of Washington Animal Care and Use Committee.

### Subjects

Experimentally naive male Long-Evans rats (250–300 g) were individually housed in a standard polycarbonate cage, equipped with feeder and water bottle, in a climate-controlled vivarium (on a 12-h light:dark cycle, lights off at 7 AM). All test procedures were conducted during the dark phase of the cycle when rats are normally active. Animals were assigned to either SINGLE, DYAD or BEHAVIORAL STRESS treatment conditions (all within-subjects design) as detailed below.

### Surgery

Animals in the SINGLE and DYAD conditions were anesthetized with ketamine HC1 (30 mg/kg) and xylazine (2.5 mg/kg), head-fixed in a stereotaxic apparatus, and implanted chronically with bipolar stainless steel wire electrodes (bare tip diameter, 0.125 mm; Plastics One) bilaterally in the basolateral nucleus of the amygdala (BLA; from Bregma: −2.8 mm posterior, 5.2 mm lateral, 8.4 mm ventral) and a dual guide cannula (1.5 mm center-to-center distance, Plastics One) in the (mPFC; from Bregma: 2.7 mm anterior, 0.5 mm lateral, 4.1 mm ventral). Animals were adapted to daily handling during the 5–7 day postoperative recovery period.

### Object Recognition (OR) Apparatus

Behavioral testing took place inside a square arena (57 × 57 × 59 cm high; constructed of white fiberboard) illuminated indirectly by an incandescent lamp and with a constant white noise (60 dB) background. An ultra-digital wireless camera (LW2101; Lorex Technology Inc.,) affixed over the apparatus was connected to a Sony HD DVD recorder (RDR-HX900) and a PC (in the adjacent room) to record the animal’s behavior. The ANY-maze video tracking system (Stoelting Company) was used to capture video images and track the animal’s movement (30 frames/s). Three identical sets of different objects, made of plastic, glass, metal or wood, and varied in shape and texture were used. All animals were exposed to two different types of (familiar and novel) objects simultaneously with the order of object presentations counterbalanced. To minimize the possible spatial-location influence, the familiar and novel objects were always placed in the same two corners of the arena in a counterbalanced manner.

### Procedure

The SINGLE rats underwent AMYG, mPFC, CORT (individual) and AMYG + mPFC + CORT (combined, COMB) manipulations (counterbalanced), whereas the DYAD rats underwent AMYG + mPFC, mPFC + CORT and AMYG + CORT (paired) and AMYG + mPFC + CORT (COMB) manipulations (counterbalanced). All animals were habituated to an open field chamber without any objects for 10 min per day for four consecutive days (habituation phase). Twenty-four hours after the last habituation session, animals were given 10 min to explore two identical objects placed in a familiar chamber (familiarization phase). Afterward, they received: (i) the GABA-A receptor agonist muscimol infusions into the mPFC (10 mM, 0.3 μl per side, 0.1 μl/min; see Yoon et al., 2008); (ii) an injection of CORT (3 mg/kg subcutaneous); which has been shown to increase the plasma corticosterone by four-fold (see Kim et al., 2012); (iii) electrical stimulation of the AMYG (0.5-ms pulses at 100 Hz, 60 5-s trains, 35–75-s ITIs, 100–400 μA), which produces freezing and 22-kHz ultrasonic vocalization (see Kim et al., 2012); (iv) dual combinations of i + ii, i + iii and ii + iii manipulations (DYAD condition); (v) a COMB i + ii + iii manipulation; (vi) behavioral stress (60 min restraint and tailshocks: 1 mA, 1-s, 5–115 s apart; Baker and Kim, 2002); or (vii) a homecage control (CTRL) condition. Considering the time it takes for exogenously administered CORT and muscimol to reach relatively stable levels (~30 min; Baraldi et al., 1979; Wiegert et al., 2006), mPFC muscimol and CORT drug injections were given promptly after the familiarization phase. The AMYG stimulation and STRESS treatments commenced ~30 min after the familiarization phase to match the CORT/muscimol time frame. On the next day (test phase), one object identical to the familiarization phase and the other a novel object was placed in the chamber and animals were given 5 min of exploration. After each phase, animals underwent at least two 5-min habituation phases before the next familiarization phase resumed.

### Behavioral Data Collection and Analysis

A custom-written program in QBASIC was used to quantify exploratory behavior (see Baker and Kim, 2002) from the ANY-maze video playback. In brief, manual keystrokes on the computer keyboard, by a trained “blind” observer, recorded the duration and frequency of object exploration. Exploration was scored only when the rat’s head both traversed a predefined object boundary outlined on the monitor screen and was directed toward the object. Exploration was not scored when the animal climbed on top of the object or if another part of the rat’s body touched the object (see Clark et al., 2000), which the ANY-maze video tracking system cannot reliably differentiate from exploratory behavior.

### Histology

At the completion of behavioral testing, marking lesions were made at the tips of stimulating electrodes (100 μA, 10 s) to verify the electrode placement. All rats were overdosed with Beuthanasia and perfused intracardially with 0.9% saline, followed by 10% buffered formalin. The brains were removed and stored in a 30% sucrose solution until they sank before slicing. Coronal sections (60 μm) were taken through the extent of the cannulae and electrode tracks, mounted on gelatinized slides, and stained with Prussian blue and cresyl violet dyes.

### Statistical Analyses

Results are presented as means ± SEM. All statistical analyses were performed with SPSS (version 11.0; SPSS Inc., Chicago, IL, USA). Object exploration time data were analyzed using a paired *t*-test (*p* < 0.05, two-tailed) and discrimination index (novel object exploration time-familiar object exploration time)/(novel object exploration time + familiar object exploration time), data were analyzed using one sample *t*-test (*p* < 0.05, two-tailed). A non-parametric Wilcoxon signed ranks test was used for those exploration time data that were not normally distributed. Of 33 rats used, two animals were excluded from analyses due to tailshock delivery and video recording errors.

## Results

**Figure 1:**
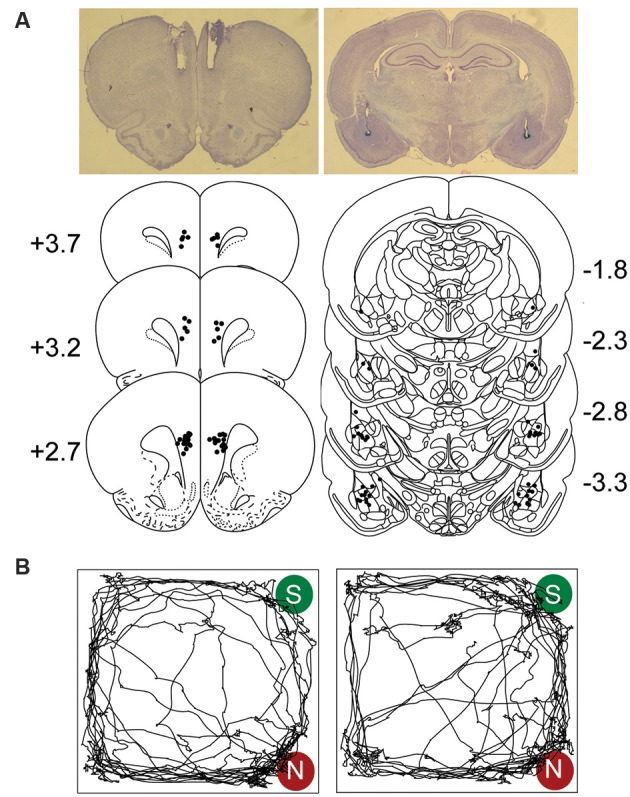
Electrodes and cannulae placements and track plots. **(A)** Photomicrograph and histological reconstruction of cannulae medial prefrontal cortex (mPFC, each dot represents the tips of the guide cannulae) and stimulating electrodes amygdala (BLA) implantations. **(B)** Representative visit maps from CTRL (left) and COMB treatments (right) during the sample (S) vs. novel (N) object test session. See text for object exploration criteria.

Figure 1A shows the placements of guide cannulae and stimulating electrodes aimed at the mPFC and BLA regions, respectively. Representative visit maps recorded during test sessions are presented in Figure 1B, showing biased (CTRL; *left*) and unbiased (COMB; *right*) exploration towards the novel object location.

**Figure 2:**
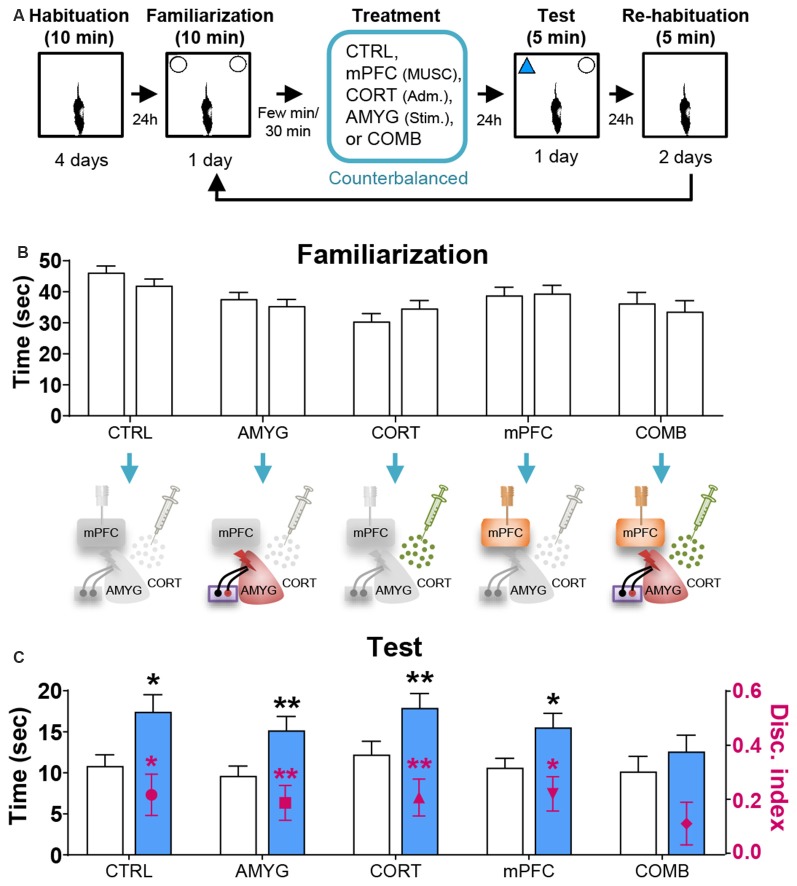
Effects of mPFC inactivation, CORT administration, and amygdalar stimulation on object recognition (OR) memory. **(A)** Behavior and treatment procedures. **(B)** Mean time in seconds (± SEM) that animals subjected to CTRL, AMYG, CORT, mPFC, and COMB treatments spent exploring two identical objects during the familiarization phase. **(C)** Mean time in seconds that different treatment animals spent exploring novel vs. familiar objects and the mean value of discrimination index during the first 2 min of the test phase. *p < 0.05 and **p < 0.01, respectively.

**Figure 3:**
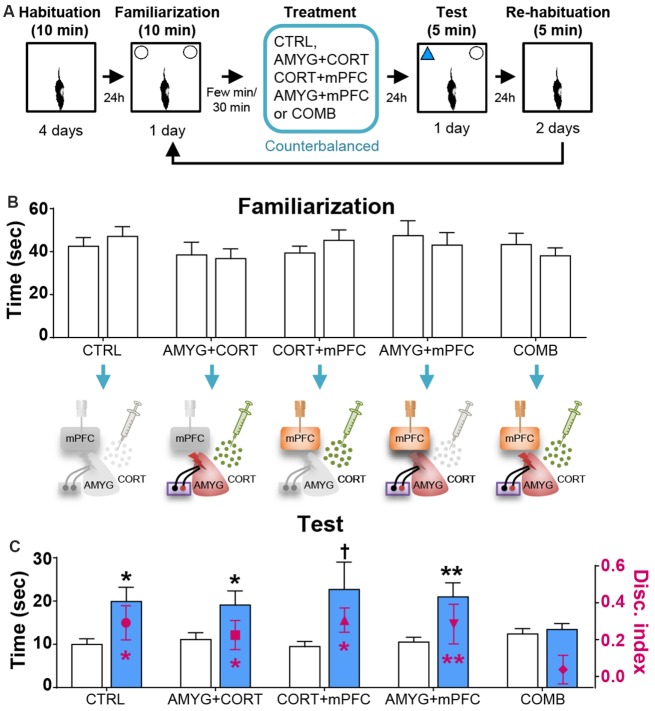
Effects of dyad treatments on OR memory. **(A)** Behavior and treatment procedures. **(B)** Mean time in seconds (± SEM) that CTRL, AMYG + CORT, CORT + mPFC, AMYG + mPFC, and COMB treatment animals spent exploring two identical objects during the familiarization phase. **(C)** Mean time in seconds that different treatment animals spent exploring novel vs. familiar objects and the mean value of discrimination index during the first 2 min of the test phase. *p < 0.05 (*t*-test), **p < 0.01 (*t*-test), and ^†^*p* < 0.05 (Wilcoxon signed ranks test), respectively.

**Figure 4:**
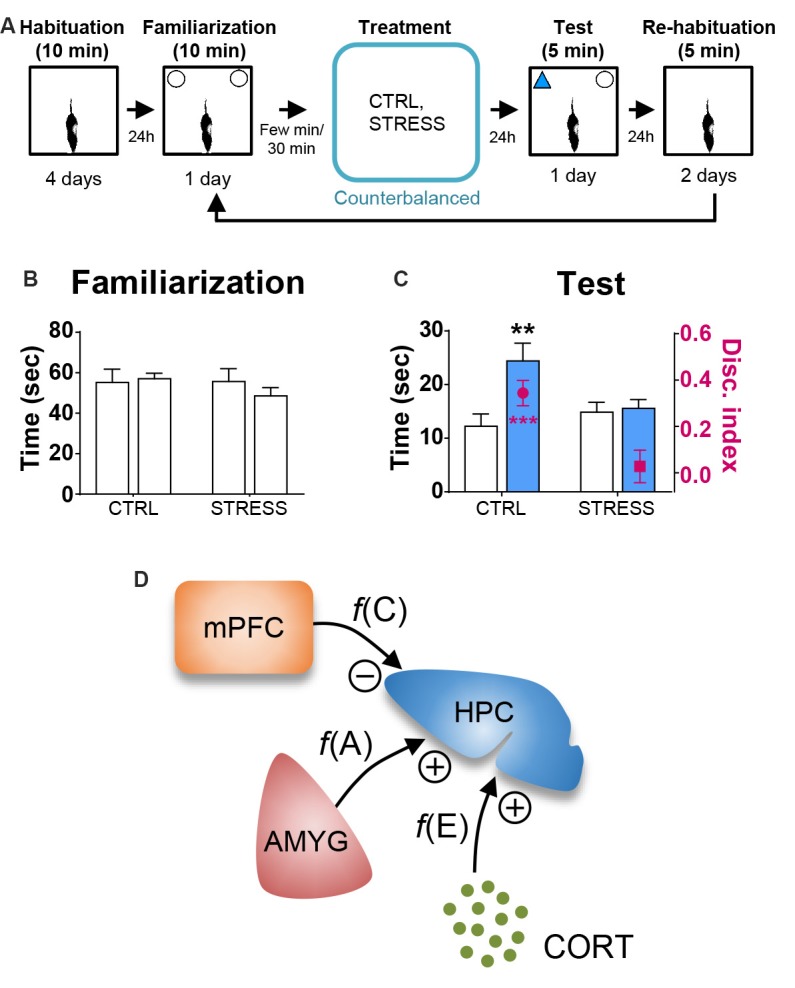
Behavioral stress effects on OR memory. **(A)** Behavioral procedure. **(B)** Mean time in seconds (± SEM) that CTRL and STRESS treatment animals spent exploring two identical objects during the familiarization phase. **(C)** Mean time in seconds that CTRL and STRESS treatment animals spent exploring novel vs. familiar objects and the mean value of discrimination index during the first 2 min of the test phase. **p < 0.01 and ***p < 0.001. **(D)** A systems-level model of stress comprising of CORT, AMYG and mPFC interaction. The model posits that the CORT, AMYG and mPFC mediate the functions of excitability *f(E)*, aversiveness *f(A)*, and controllability *f(C)*, respectively, and that CORT and AMYG exert excitatory stress influences while mPFC exerts inhibitory stress influence on the hippocampus (HPC). Adapted from references Kim and Diamond (2002); Kim and Haller (2007) and Kim et al. (2015).

Table 1 shows the mean ± SEM object exploration time (in seconds) in the arena during the familiarization (two identical objects; 10 min) and test (familiar vs. novel objects; 5 min) phases for all animals. None of the treatments (Figures 2A, 3A, 4A) reliably altered the amounts of time exploring the two identical objects (SINGLE: *t*’s < 1.3, *p*’s > 0.2, paired *t*-test, Figure 2B; DYAD: *t*’s < 1.313, *p*’s > 0.225, Figure 3B; STRESS: *t*’s < 1.5, *p*’s > 0.2, paired *t*-test; Figure 4B). This suggests that there were no residual effects of the surgery and repeated testing (following treatments) on the animals’ sensory, motor, and motivational systems for exploring objects.

**Table 1:** Mean total exploration time in seconds (±SEM) animals spent exploring two objects during the familiarization and test phases.

Treatment	Familiarization	Test
**Single**
CTRL	86.1 ± 11.20	55.8 ± 4.48
AMYG	73.0 ± 6.48	50.1 ± 4.64
CORT	65.0 ± 7.56	54.3 ± 6.88
mPFC	78.2 ± 8.57	49.3 ± 4.88
AMYG + CORT + mPFC	69.8 ± 9.16	39.7 ± 6.32
**Dyad**		
CTRL	89.6 ± 7.49	52.4 ± 5.77
AMYG + CORT	75.3 ± 9.66	58.6 ± 9.53
CORT + mPFC	84.6 ± 6.85	58.2 ± 8.37
AMYG + mPFC	90.5 ± 9.36	66.1 ± 5.79
AMYG + CORT + mPFC	81.4 ± 6.95	47.2 ± 5.42
**Behavioral stress**		
CTRL	112.2 ± 8.28	69.2 ± 9.19
Stress	104.3 ± 8.98	53.4 ± 7.86

Based on the literature, the first 2 min of exploration time during the test phase, before habituation to the novel object transpired, gives a reliable measure of OR memory (Dix and Aggleton, 1999; Barker et al., 2007). We analyzed this testing period and found that the control and all individual treatment rats (in SINGLE condition) spent significantly more time exploring the novel than familiar object (CTRL, *t*_(14)_ = 2.614, *p* < 0.05; AMYG, *t*_(14)_ = 3.059, *p* < 0.01; CORT, *t*_(14)_ = 3.004, *p* < 0.01; mPFC, *t*_(14)_ = 2.472, *p* < 0.05; Figure 2C). The analysis of discrimination index yielded the same results (all *t*’s > 2.839, *p*’s < 0.05; Figure 2C). DYAD treatments also did not impair memory performance during the test phase as shown by time (CTRL, *t*_(8)_ = 3.111, *p* < 0.05; AMYG + CORT, *t*_(8)_ = 2.403, *p* < 0.05; CORT + mPFC, *Z* = 2.192, *p* < 0.05; AMYG + mPFC, *t*_(8)_ = 3.693, *p* < 0.01; Figure 3C) and discrimination index (all *t*’s > 2.631, *p*’s < 0.05; Figure 3C). In both SINGLE and DYAD conditions, however, the COMB (AMYG + mPFC + CORT) treatment rats did not demonstrate a preference for the novel object over the familiar object (*t*’s < 1.397, *p*’s > 0.18, Figure 2C; *t*’s < 0.519, *p*’s > 0.61, Figure 3C). The effects of AMYG + mPFC + CORT (COMB) treatments on OR memory performance were comparable to that of uncontrollable behavioral stress. While the CTRL rats spent more time exploring the novel object than the previously explored object (time: *t*_(6)_ = 5.485, *p* < 0.01; discrimination index: *t*_(6)_ = 6.300, *p* < 0.001; Figure 4C), the STRESS animals did not exhibit preference for the novel object over the familiar object (time: *t*_(6)_ = 0.340, *p* = 0.745; discrimination index: *t*_(6)_ = 0.389, *p* = 0.711), as previously reported (Baker and Kim, 2002). These results indicate that combined, but not individual or dual, treatments of AMYG stimulation, CORT injection, and mPFC inhibition are sufficient to mimic impairing effects of stress on OR memory in naïve rats.

## Discussion

In recent decades, the mainstream approaches to investigating stress effects on the brain functions have been to relate the levels and activities of particular hormones, such as glucocorticoids, from the adrenal gland (McEwen and Sapolsky, 1995), peptides, such as corticotropin-releasing factor (CRF), from the hypothalamic paraventricular nucleus (Heinrichs et al., 1995), or neurotransmitters, such as serotonin, from the DRN (Maier and Watkins, 2005), directly to stress. As these strategies are experimentally tractable to both *in vitro* and *in vivo* analyses, they have generated a wealth of information putatively in relation to stress (Schaaf et al., 2000; Groc et al., 2008). However, whether a single biochemical system can accurately reflect the multifaceted neural-cognitive-behavioral characteristics of stress needs to be logically questioned (e.g., Kim et al., 2015). Consistent with the view that no single biochemical substance responds uniquely to stress and, thus, none is likely to be a sufficient causal factor of stress, the present findings show that the systemic administration of corticosterone, which yields four-fold increases in the circulating corticosterone level (Kim et al., 2012), failed to influence 24-h delay OR memory, a putative hippocampal-dependent memory task (e.g., Clark et al., 2000; Baker and Kim, 2002; Broadbent et al., 2010; Zhao et al., 2012; Mello-Carpes and Izquierdo, 2013; but see Mumby, 2001). As alluded previously, sex, environment enrichment and exercise all significantly elevate corticosterone levels, but none have been found to impair hippocampal memory functions (for a recent review, see Kim et al., 2015). Environment enrichment and exercise, if anything, enhance dendritic arborization, synaptogenesis, and neurogenesis in the hippocampus, which are opposite effects of stress (e.g., Schoenfeld and Gould, 2012). Furthermore, if glucocorticoids are the main contributing factors in the mediation of stress effects, where low/high levels facilitate/impede hippocampal functions, then removing glucocorticoids during stress and directly applying glucocorticoids in the absence of behavioral stress should preclude and produce stress effects, respectively. However, there are several behavioral, synaptic plasticity, and neural activity data from animal studies inconsistent with this simple curvilinear chemical level-stress effect notion (Kim et al., 2015).

In the present study, the OR memory performance was also unaffected by inhibition of the mPFC, a structure implicated in the top-down controllability of stressor (Amat et al., 2005; Dalley et al., 2011) or stimulation of the amygdala, a structure concerned with affective responses. However, unlike corticosterone injections and intra-mPFC muscimol infusions, which did not elicit visible distress behaviors, the 60 min intermittent electrical stimulations of the amygdala evoked robust freezing and 22 kHz ultrasonic vocalization behaviors in rats (Kim et al., 2012). The same amygdalar stimulation was also found to alter the firing properties of the hippocampal CA1 place cells (Kim et al., 2012), akin to behavioral stress (Kim et al., 2007). This suggests that amygdalar stimulation-induced alterations of place cells are not critically connected to the OR memory functioning, at least not when the objects are placed on the constant locations in the open-field arena. In contrast to negative findings with individual/dyad corticosterone, mPFC, and amygdala manipulations, the combination of all three treatments was sufficient to impede OR memory performance, comparable to uncontrollable stress. These effects on the OR memory are unlikely due to extraneous factors, such as alterations in the motor and/or motivational systems, because the combined treatments occurred after the animals have already explored two identical objects during the familiarization phase and because any non-specific effects associated with the treatments would have dissipated by the time of the novelty preference test the next day.

The null effects of corticosterone treatment on the OR memory performance are inconsistent with the prevalent view where stress and glucocorticoids are often considered interchangeable (Sapolsky et al., 1986; de Quervain et al., 1998; McEwen, 1999; Yehuda, 2009), when the main function of glucocorticoids is to regulate glucose homeostasis not exclusive to stress but to various psychological and physical events (Nicolaides et al., 2000; Kuo et al., 2015). The present findings are instead more in line with the notion that stress involves three basic psychological factors of excitability/arousal, aversiveness, and uncontrollability, which correspond to biological substrates of elevated levels of glucocorticoids, increased activity in the amygdala, and decreased activity in the mPFC, respectively (Kim and Diamond, 2002; Kim and Haller, 2007; Kim et al., 2015; Figure 4D). These psychological-biological designations are consistent with the evidence that the HPA-axis activity correlates with excitability/arousal (de Quervain et al., 1998; Gutteling et al., 2005; Yehuda et al., 2005), amygdala inactivation/stimulation reduces/evokes aversive responses (Henke, 1990; Helmstetter, 1993; Adamec et al., 1999), and mPFC activity correlates with behavioral controllability while mPFC damage results in the loss of behavioral control (Herry and Garcia, 2002; Milad and Quirk, 2002; Izquierdo et al., 2006; Maier et al., 2006; Radley et al., 2006). The proposed stress model, based on clearly defined psychological constructs and physiologically anchored, offer predictability and testability, *via* manipulating corticosterone, amygdala, and mPFC in conjunction with behavioral stress. Future studies will also need to determine whether the combined treatments influence other aspects of hippocampal functions (e.g., LTP, dendritic arborization, neurogenesis), identify the specific region of the mPFC (e.g., prelimbic, infralimbic and cingulate cortices) and cell type that contribute to the stress response (e.g., *via* optogenetic and chemogenetic tools) and its pathway to the hippocampus (e.g., most likely indirect projections *via* entorhinal cortex or nucleus reuniens), and how corticosterone, amygdala and mPFC inputs integrate to affect the hippocampus. The systems-level approaches, rather than focusing on singular chemical systems, are likely to lead to a better understanding of how stress affects the brain and cognition.

## Data Availability

The datasets generated for this study are available on request to the corresponding author.

## Ethics Statement

The animal study was reviewed and approved by The University of Washington Animal Care and Use Committee.

## Author Contributions

EK and JK designed the research, analyzed the data, wrote the manuscript and contributed to the preparation of the manuscript. EK performed research. All authors revised and approved the final version of the manuscript.

## Conflict of Interest Statement

The authors declare that the research was conducted in the absence of any commercial or financial relationships that could be construed as a potential conflict of interest.
